# Intestinal organoids in inflammatory bowel disease: advances, applications, and future directions

**DOI:** 10.3389/fcell.2025.1517121

**Published:** 2025-05-12

**Authors:** Jianzhen Ren, Silin Huang

**Affiliations:** Department of Gastroenterology, South China Hospital, Medical School, Shenzhen University, Shenzhen, China

**Keywords:** intestinal organoids, inflammatory bowel disease, application, progress, new therapies

## Abstract

Inflammatory bowel disease (IBD), characterized by chronic gastrointestinal inflammation, is a significant global health challenge. Traditional models often fail to accurately reflect human pathophysiology, leading to suboptimal treatments. This review provides an overview of recent advancements in intestinal organoid technology and its role in IBD research. Organoids, derived from patient-specific or pluripotent stem cells, retain the genetic, epigenetic, and structural characteristics of the native gut, allowing for precise modeling of key aspects of IBD. Innovations in CRISPR editing, organoid-microbe co-cultures, and organ-on-a-chip systems have enhanced the physiological relevance of these models, facilitating drug discovery and personalized therapy screening. However, challenges such as vascularization deficits and the need for standardized protocols remain. This review underscores the need for interdisciplinary efforts to bridge the gap between models and the complex reality of IBD. Future directions include the development of scalable vascularized models and robust regulatory frameworks to accelerate therapeutic translation. Organoids hold promise for unraveling IBD heterogeneity and transforming disease management.

## Graphical Abstract

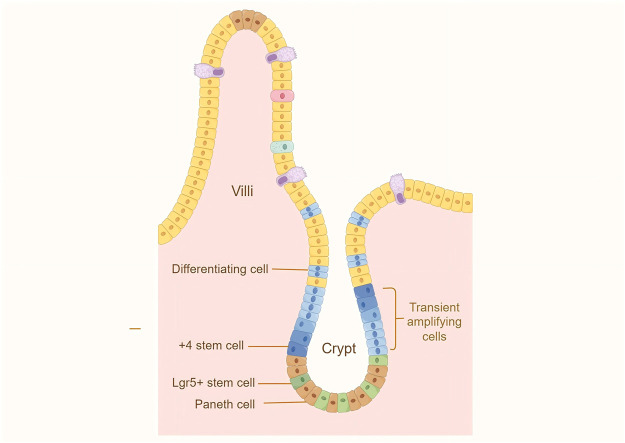

## 1 Introduction

Inflammatory bowel disease (IBD), which includes Crohn’s disease (CD) and ulcerative colitis (UC), is increasingly becoming a significant global health concern, marked by changing epidemiological trends. Notably, there has been a marked upsurge in incidence rates in newly industrialized regions across Asia and South America. Meanwhile, in Western countries, prevalence figures have already surpassed the 0.3% threshold (Kaplan and Windsor, 2021; Ananthakrishnan et al., 2020). The chronic and relapsing nature of IBD, compounded by complications such as intestinal fibrosis and an elevated risk of colorectal cancer, presents formidable clinical challenges (Lewis et al., 2023; Loftus, 2021). Traditional research models, encompassing animal studies and 2D cell cultures, often fall short in terms of translational relevance. This is largely due to interspecies physiological differences and their inability to accurately replicate the intricate architecture of the human intestine and its immune interactions (DeHaan and Huang, 2020). For example, murine models are incapable of fully replicating the genetic diversity and microbial dynamics characteristic of human IBD. Similarly, 2D epithelial monolayers fail to capture the multicellular complexity inherent to the gut microenvironment (Kollmann et al., 2023). These limitations have highlighted the pressing need for more advanced models that can effectively bridge the gap between preclinical research findings and their clinical applications.

**FIGURE 1:**
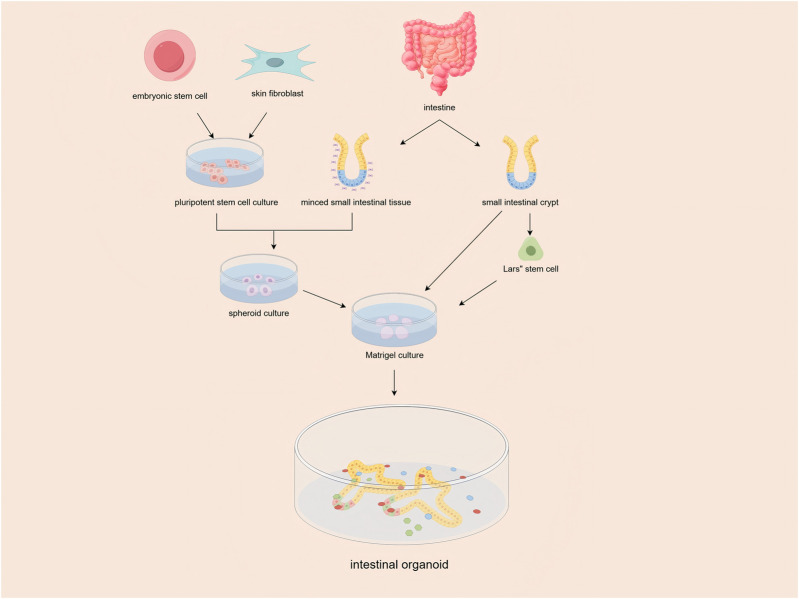
Isolated intestinal stem cells (ISCs) or tissues containing ISCs were embedded in Matrigel and maintained in culture medium supplemented with essential niche factors. the ISCs self-organized into intestinal organoids, forming 3-dimensional structures with crypt-like buds.

Since their inception in 2009 (Sato et al., 2009), intestinal organoids, which are self-organizing 3D structures derived from adult stem cells or pluripotent stem cells, have brought about a revolutionary transformation in IBD research. Initially developed by embedding Lgr5+ intestinal stem cells in Matrigel (Figure 1), this technology has since evolved to include patient-derived organoids (PDOs), thereby enabling highly specific disease modeling (Cable et al., 2022). Several key advancements have further bolstered the potential of organoids. The integration of CRISPR-Cas9 technology has allowed for precise genetic manipulation, while the development of organoid-microbiome co-culture systems has facilitated in-depth studies of host-microbe interactions (Li, 2021; de Poel et al., 2023). More recently, the advent of organ-on-a-chip platforms has added another layer of sophistication by incorporating mechanical forces and immune components, thereby enhancing the physiological relevance of these models (Gjorevski et al., 2022). Collectively, these breakthroughs have established organoids as a cornerstone in the study of intestinal development, pathophysiology, and personalized therapeutics.

The unique strengths of intestinal organoids are particularly noteworthy. They have the remarkable ability to preserve patient-specific genetic and epigenetic signatures while accurately replicating the crypt-villus architecture and cellular diversity of the native gut (Ojo et al., 2022). Unlike their traditional counterparts, organoids maintain functional epithelial barrier properties, mucus secretion, and drug-metabolizing enzymes, rendering them highly suitable for both mechanistic studies and drug screening (Schulte et al., 2019). For instance, organoids derived from IBD patients have not only identified dysregulated Wnt/β-catenin signaling in epithelial repair but have also demonstrated individualized responses to biologics such as vedolizumab (Jelinsky et al., 2023; Cao et al., 2023). Against this backdrop, this review aims to delve into how organoid technology is reshaping IBD research. It will explore the technology’s role in elucidating disease mechanisms and accelerating the progress of precision medicine.

## 2 Technical foundations of intestinal organoids

### 2.1 Intestinal organoid culture techniques: key methods

The foundation of intestinal organoid technology lies in the isolation and maintenance of Lgr5+ intestinal stem cells (ISCs), which are capable of self-renewal and multilineage differentiation. Typically, these cells are embedded in a three-dimensional extracellular matrix (ECM), such as Matrigel, to replicate the crypt-villus niche characteristic of the native gut. However, recent advancements have seen a shift towards synthetic hydrogels as an alternative to animal-derived matrices. These synthetic materials offer tunable stiffness and reduced batch variability, which are crucial for consistent and reproducible results. For example, Hushka et al. (2024) developed fully synthetic hydrogels that enhance crypt formation by modulating matrix viscoelasticity, thereby improving structural fidelity and reproducibility. In a similar vein, Hernandez-Gordillo et al. (2020) engineered synthetic scaffolds that support primary human intestinal enteroids without the need for biological matrices, thus enabling standardized culture conditions. Innovations in bioengineering have further refined spatial control, with Cambra et al. (2022) introducing a triple-decker sandwich system for long-term live imaging and uniform perturbations. This system facilitates the precise analysis of organoid morphogenesis. Additionally, photodegradable hydrogels, such as those developed by Yavitt et al. (2023), allow for on-demand matrix remodeling to guide crypt patterning. Collectively, these approaches highlight a shift towards scalable and defined platforms that preserve stem cell functionality while minimizing reliance on undefined substrates (Kim et al., 2022; Xu et al., 2023).

### 2.2 Classification of intestinal organoid models: normal vs. disease-specific

Intestinal organoids can be broadly classified into two categories: normal and disease-specific models. Normal organoids, derived from healthy tissues, are designed to recapitulate the homeostatic processes of epithelial turnover and crypt-villus dynamics. For instance, transcriptomic profiling conducted by Criss et al. (2021) identified Wnt signaling and metabolic pathways as key regulators of epithelial homeostasis in human enteroids. In contrast, disease-specific organoids are engineered to model the pathological mechanisms underlying various intestinal disorders. Patient-derived ulcerative colitis (UC) organoids, for example, exhibit impaired barrier protein expression and dysregulated differentiation, thereby mirroring the *in vivo* disease phenotypes observed in clinical settings (Aslam et al., 2020). Similarly, familial adenomatous polyposis (FAP) organoids, generated by Laborde et al. (2025), reveal early tumorigenic events driven by APC mutations, highlighting the altered dynamics of stem cells in this context. Infectious disease models, such as those involving Cryptosporidium parvum-infected enteroids, allow researchers to dissect the mechanisms of pathogen-induced epithelial damage and host immune responses (Lamisere et al., 2022). These models are not only valuable for basic research but also serve as platforms for drug screening. For example, Lucafò et al. (2022) demonstrated the potential for personalized therapy testing using IBD PDOs, while Patil et al. (2025) identified 2′-fucosyllactose as a potent inhibitor of norovirus replication. These applications underscore the versatility of organoids in bridging the gap between basic research and clinical translation, thereby emphasizing their potential in disease modeling (Ojo et al., 2022; Poplaski et al., 2023).

## 3 Applications of intestinal organoids in IBD research

### 3.1 Disease mechanism exploration

Intestinal organoids have emerged as powerful tools for dissecting the genetic and epigenetic underpinnings of inflammatory bowel disease (IBD). For instance, single-cell RNA sequencing of pediatric Crohn’s disease (CD) PDOs has revealed transcriptional signatures linked to epithelial cell dysfunction and immune dysregulation. This research highlights the role of the TNFAIP3 and NOD2 pathways in disease progression (Elmentaite et al., 2020). Epigenetic alterations, particularly DNA methylation patterns, have also been implicated in IBD pathogenesis. Howell et al. (2018) demonstrated that DNA methylation profiles in intestinal epithelial cells (IECs) from pediatric IBD patients correlate with disease subtypes and clinical outcomes, suggesting that epigenetic regulation is a key driver of mucosal inflammation. Further, Fazio et al. (2022) identified DNA methyltransferase 3A (DNMT3A) as a critical regulator of epithelial barrier integrity and regeneration in colitis models, linking epigenetic modifications to impaired wound healing in IBD.

Another focal area of research is the interaction between epithelial barrier dysfunction and immune microenvironment dynamics. Studies using CD PDOs have shown persistent defects in tight junction proteins (e.g., ZO-1) and increased permeability, even in remission states. These findings underscore the role of epithelial-immune crosstalk in sustained inflammation (Meir et al., 2020). Additionally, Martínez-Sánchez et al. (2023) revealed that RAC1-dependent cytoskeleton remodeling in IECs modulates cell shedding and barrier integrity during inflammation. This provides mechanistic insights into how epithelial mechanics influence immune activation. Collectively, these findings emphasize the utility of organoids in modeling the complex interplay between genetic, epigenetic, and microenvironmental factors in IBD.

### 3.2 Translational research

Intestinal organoids are not only valuable for understanding disease mechanisms but are also revolutionizing drug discovery and personalized therapy for IBD. High-throughput screening platforms using PDOs have enabled rapid evaluation of drug efficacy. For example, Buttó et al. (2020) utilized Crohn’s disease-like ileitis organoids to test anti-inflammatory compounds, identifying Wnt agonists as potential candidates to restore stem cell niche function. Similarly, Woznicki et al. (2021) demonstrated that TNF-α and IFN-γ synergistically induce epithelial cell death via caspase-8-JAK1/2-STAT1 pathways. This model is now used to screen caspase inhibitors for barrier protection.

Personalized medicine applications are equally promising. Karakasheva et al. (2023) showed that colonoids from IBD patients retain disease-specific transcriptomic signatures, enabling tailored drug testing based on individual molecular profiles. Lucafò et al. (2022) highlighted cases where therapy responses in PDOs predicted clinical outcomes, such as corticosteroid efficacy in restoring barrier function in CD-derived models. Furthermore, organoid-microbe co-cultures and organ-on-a-chip systems are being integrated to mimic host-microbiome interactions. This integration aids in the development of microbiome-targeted therapies (Beaurivage et al., 2020; Shin et al., 2020). These advances underscore the potential of organoids to bridge preclinical research and individualized clinical care in IBD.

## 4 Recent advances and breakthroughs

### 4.1 Organoid-microbe co-culture systems to decipher microbiome roles in IBD

Recent advancements in organoid-microbe co-culture systems have significantly enhanced our understanding of microbiome-host interactions in IBD pathogenesis. For instance, Beaurivage et al. (2020) pioneered a human primary gut-on-a-chip model that integrates microbial antigens to mimic inflammatory processes. Their work revealed how dysbiotic microbiota disrupt epithelial barrier integrity and trigger immune activation. Similarly, Shin et al. (2020) developed a physiodynamic mucosal interface-on-a-chip that co-cultures patient-derived intestinal organoids with commensal or pathogenic bacteria. This innovative approach demonstrated species-specific modulation of epithelial tight junction proteins and cytokine profiles in IBD. Building on these findings, Ahn et al. (2023) further highlighted the utility of organoid-microbe systems in identifying microbial metabolites, such as butyrate, that restore epithelial homeostasis by regulating immune cell recruitment. Notably, Ballerini et al. (2025) combined fecal microbiota transplants from IBD patients with gut-on-a-chip platforms to predict personalized responses to microbiome-targeted therapies, effectively bridging translational gaps. These models not only recapitulate complex host-microbiome crosstalk but also enable high-throughput screening of probiotics or antimicrobial agents to restore microbial balance in IBD (Kumari et al., 2025).

### 4.2 CRISPR gene editing innovations in intestinal organoid disease modeling

Parallel to advancements in microbiome research, CRISPR-Cas9-mediated genome editing has revolutionized disease modeling using intestinal organoids. Beumer et al. (2022) established a biobank of CRISPR-engineered organoids to identify host factors critical for coronavirus infection. Their work showcased the adaptability of CRISPR technology for studying viral entry mechanisms in IBD-associated epithelial dysfunction. Hansen et al. (2023) employed a CRISPR-Cas9 screen in human intestinal organoids to uncover transcriptional regulators of epithelial maturation, linking NOD2 mutations to impaired Paneth cell differentiation in Crohn’s disease. Skoufou-Papoutsaki et al. (2023a) optimized ribonucleoprotein-based CRISPR editing in IBD PDOs, enabling precise correction of disease-associated variants (e.g., IL23R) while preserving native epigenetic states. Furthermore, Martinez-Silgado et al. (2022) demonstrated the differentiation of CRISPR-modified pluripotent stem cells into functional intestinal organoids, providing a scalable platform to model genetic subtypes of IBD. Collectively, these breakthroughs highlight CRISPR-edited organoids as indispensable tools for dissecting genotype-phenotype relationships and developing gene therapies.

### 4.3 Advancements in gut-on-a-chip technology

In addition to co-culture systems and gene editing, organ-on-a-chip (OOC) technologies have emerged as powerful platforms to enhance the physiological relevance of intestinal organoid models. Beaurivage et al. (2020) integrated human intestinal organoids into microfluidic chips with peristalsis-mimicking shear stress, revealing mechanosensitive pathways that regulate epithelial repair in colitis. Shin et al. (2020) advanced this concept by developing a multi-layered gut-on-a-chip that incorporates immune cells and microbiota, enabling real-time analysis of barrier dysfunction and neutrophil infiltration in IBD. An et al. (2025) highlighted the application of OOC systems in pharmacokinetic studies, where vascularized intestinal chips predict drug absorption and toxicity with human-specific accuracy. Additionally, Carvalho et al. (2024) designed a modular LEGO-like microfluidic platform to assemble heterogeneous organoid-immune cell co-cultures, facilitating personalized testing of biologic therapies. These innovations underscore the potential of OOC systems to bridge *in vitro* and *in vivo* studies, offering unprecedented resolution in modeling IBD complexity (Özkan et al., 2024).

## 5 Current challenges and limitations

### 5.1 Technical limitations: vascularization, immune component deficiencies, and long-term culture stability

Despite significant advancements, intestinal organoids still face several technical limitations that hinder their full potential in modeling complex physiological processes in IBD. A critical barrier is the lack of vascularization. Current 3D cultures primarily rely on passive diffusion for nutrient supply, which cannot replicate the dynamic vascular network essential for immune cell trafficking and sustained tissue viability. Recent studies have highlighted that vascularization is crucial for modeling immune-epithelial interactions in IBD, as the absence of endothelial cells limits the recruitment of immune components such as macrophages and T cells (Wen et al., 2024). Additionally, long-term culture stability is compromised by the gradual loss of stemness and structural integrity, particularly in disease-specific organoids derived from IBD patients (Lee et al., 2023). While advanced strategies, such as organoid-on-a-chip systems, aim to integrate microfluidic channels to mimic vascular flow, issues of scalability and reproducibility remain unresolved (Liu et al., 2025). Furthermore, immune component deficiencies hinder the study of IBD’s inflammatory microenvironment, as most organoid models lack resident immune cells or microbiota interactions (Kistemaker et al., 2025). Addressing these limitations will require interdisciplinary approaches that combine bioengineering and immunology to develop vascularized, immune-competent organoids.

### 5.2 Biological complexity: discrepancies between organoids and *in vivo* microenvironments

Another significant challenge is the biological complexity of the gut microenvironment, which is often oversimplified in organoid models. While intestinal organoids can effectively recapitulate epithelial architecture, they often overlook the multicellular complexity of the gut microenvironment. For instance, organoids lack the diverse stromal cell populations, including fibroblasts and pericytes, which play crucial roles in modulating epithelial repair and immune responses in IBD (Parente et al., 2024). Single-cell sequencing has revealed significant transcriptional differences between organoid-derived epithelial cells and their *in vivo* counterparts, particularly in pathways related to hypoxia and microbial sensing (Zhao et al., 2024). Moreover, the absence of microbiota in standard cultures limits their utility for studying host-microbe interactions, a cornerstone of IBD pathogenesis (Sugihara et al., 2025). Recent efforts to co-culture organoids with microbial communities or immune cells have partially addressed these gaps but face challenges in maintaining stable symbiosis or inflammatory states (Günther et al., 2022). These discrepancies underscore the need for multi-omics integration and advanced co-culture systems to bridge the gap between reductionist models and physiological complexity.

### 5.3 Standardization and clinical translation barriers

The translational potential of intestinal organoids is further hampered by a lack of standardized protocols and validation frameworks. Variations in matrix composition, growth factors, and stem cell sources lead to inconsistent organoid phenotypes across laboratories, complicating data comparison (Zhou et al., 2023). For example, differences in extracellular matrix stiffness significantly alter organoid morphology and drug response, raising concerns about reproducibility (Lingard et al., 2024). Clinical translation is also hindered by regulatory uncertainties, as organoid-based therapies require rigorous validation of safety and efficacy, which existing preclinical models inadequately address (Benboubker et al., 2024). Additionally, scaling organoid production for high-throughput drug screening or personalized medicine demands automated platforms, yet current methods remain labor-intensive and costly (Zieger et al., 2024). Collaborative initiatives, such as the ISSCR standards for stem cell research, provide guidelines but need broader adoption to ensure harmonization (Ludwig et al., 2023). Overcoming these barriers will demand global consensus on quality control metrics and investment in scalable technologies.

## 6 Future perspectives

### 6.1 Technical improvements: vascularization and immune cell co-culture

To fully harness the potential of intestinal organoids in modeling inflammatory bowel disease (IBD), it is imperative to integrate vascular networks and immune components into these models. This integration is essential for accurately recapitulating the complex microenvironment of IBD. Recent advancements in organoid vascularization have shown promising results. For example, Landau et al. (2024a) developed bioengineered vascularized intestinal organoids using 3D bioprinting, which demonstrated enhanced nutrient delivery and maturation of epithelial layers. Similarly, Orge et al. (2024) engineered vascular units within organoids through hybrid hydrogel systems, improving oxygen diffusion and mimicking physiological angiogenesis.

Addressing immune interactions is equally critical. Co-culture models that incorporate macrophages, T cells, or microbiota-derived signals have emerged as a solution. Zhou et al. (2022) established cholangiocarcinoma organoids co-cultured with effector T cells to study immune cytotoxicity, a method that could be adapted for IBD to explore T cell-epithelial crosstalk. Additionally, Flores-Flores-Torres et al. (2023) designed bioprinted tumor-immune organoids with tumor-infiltrating lymphocytes, highlighting the potential to model IBD-specific immune responses. Future efforts should prioritize the development of standardized protocols for immune cell integration and vascular maturation, thereby bridging the gap between *in vitro* models and *in vivo* complexity.

### 6.2 Multi-omics integration and AI-driven data analysis

The integration of multi-omics technologies and artificial intelligence (AI) is poised to revolutionize IBD research using organoids. Single-cell RNA sequencing (scRNA-seq) and spatial transcriptomics now enable high-resolution mapping of epithelial-immune interactions in disease-specific organoids. For instance, Liu et al. (2024) combined scRNA-seq with patient-derived lung cancer organoids to dissect immune infiltration patterns, a framework that could be applied to IBD for identifying dysregulated pathways. Machine learning (ML) algorithms further enhance data interpretation. Du et al. (2023) employed AI to analyze morphological features of organoids, automating quality control and phenotypic classification. Similarly, Gonzalez-Ferrer et al. (2024) developed a deep-learning tool, SIMS, for label-free cell-type identification in organoid transcriptomes, accelerating biomarker discovery. Multi-omics integration, such as SCAR (Single-cell and Spatially-resolved Cancer Resources), provides a comprehensive atlas of cellular heterogeneity and metabolic states (Deng et al., 2024). Future directions include AI-driven predictive modeling for drug response and patient stratification, leveraging organoid-derived multi-omics datasets to uncover IBD-specific therapeutic targets.

### 6.3 Translational pathways: from lab to clinic

Translating organoid research into clinical applications is the next frontier. This translation requires robust validation and scalable methodologies. PDOs are increasingly used for personalized drug screening. For example, Matsumoto et al. (2024) correlated pancreatic cancer organoid drug responses with clinical outcomes, a strategy that could be adapted for IBD therapeutics. Bulcaen et al. (2024) showcased prime editing in cystic fibrosis organoids to correct mutations, underscoring the potential for CRISPR-based therapies in IBD genetic subtypes. Clinical trials are now incorporating organoid-guided designs. For instance, Wai et al. (2025) utilized liver organoids to predict outcomes of Kasai portoenterostomy, a model that could inform IBD surgical interventions. However, challenges remain in standardizing organoid production and establishing biobanks. Initiatives like the PROMOLE study (Bironzo et al., 2022) emphasize the need for multicenter collaborations to validate organoid-based biomarkers. Future steps include developing regulatory frameworks for organoid use in clinical trials and integrating organoid data with electronic health records to refine precision medicine approaches.

## 7 Conclusion

In summary, intestinal organoids have emerged as a transformative tool in IBD research, offering unprecedented insights into disease mechanisms and therapeutic strategies. These 3D models, derived from patient-specific or pluripotent stem cells, provide a unique platform for dissecting the complex interplay between genetics, epigenetics, and the gut microenvironment (Khaloian et al., 2020). Recent advancements in organoid-microbe co-cultures (Ahn et al., 2022), CRISPR gene editing Andrysiak et al. (2021), and organ-on-a-chip technologies have significantly enhanced their physiological relevance and translational potential (Carvalho et al., 2023). However, challenges remain, including the need for vascularization (Liu et al., 2024), immune component integration, and standardized protocols. Addressing these limitations will require interdisciplinary collaboration and innovation. Future directions should focus on developing scalable, vascularized models and robust regulatory frameworks to accelerate the translation of organoid research into clinical practice (Büning and Reckzeh, 2025). As we continue to unravel the heterogeneity of IBD, organoids hold great promise for advancing personalized medicine and improving patient outcomes.
